# Actinobacteria from Arctic and Atlantic deep-sea sediments—Biodiversity and bioactive potential

**DOI:** 10.3389/fmicb.2023.1158441

**Published:** 2023-03-30

**Authors:** Inês Ribeiro, Jorge T. Antunes, Diogo A. M. Alexandrino, Maria Paola Tomasino, Eduarda Almeida, Ana Hilário, Ralph Urbatzka, Pedro N. Leão, Ana P. Mucha, Maria F. Carvalho

**Affiliations:** ^1^CIIMAR - Interdisciplinary Centre of Marine and Environmental Research, University of Porto, Porto, Portugal; ^2^School of Medicine and Biomedical Sciences (ICBAS), University of Porto, Porto, Portugal; ^3^Department of Environmental Health, School of Health, Polytechnic of Porto, Porto, Portugal; ^4^Department of Biology, FCUP - Faculty of Sciences of the University of Porto, Porto, Portugal; ^5^Centre for Environmental and Marine Studies and Department of Biology, University of Aveiro, Aveiro, Portugal

**Keywords:** actinobacteria, antimicrobial, anti-cancer, anti-inflammatory, deep-sea sediments, metabarcoding

## Abstract

The deep-sea covers over 70% of the Earth’s surface and harbors predominantly uncharacterized bacterial communities. Actinobacteria are the major prokaryotic source of bioactive natural products that find their way into drug discovery programs, and the deep-sea is a promising source of biotechnologically relevant actinobacteria. Previous studies on actinobacteria in deep-sea sediments were either regionally restricted or did not combine a community characterization with the analysis of their bioactive potential. Here we characterized the actinobacterial communities of upper layers of deep-sea sediments from the Arctic and the Atlantic (Azores and Madeira) ocean basins, employing 16S rRNA metabarcoding, and studied the biosynthetic potential of cultivable actinobacteria retrieved from those samples. Metabarcoding analysis showed that the actinobacterial composition varied between the sampled regions, with higher abundance in the Arctic samples but higher diversity in the Atlantic ones. Twenty actinobacterial genera were detected using metabarcoding, as a culture-independent method, while culture-dependent methods only allowed the identification of nine genera. Isolation of actinobacteria resulted on the retrieval of 44 isolates, mainly associated with *Brachybacterium*, *Microbacterium*, and *Brevibacterium* genera. Some of these isolates were only identified on a specific sampled region. Chemical extracts of the actinobacterial isolates were subsequently screened for their antimicrobial, anticancer and anti-inflammatory activities. Extracts from two *Streptomyces* strains demonstrated activity against *Candida albicans*. Additionally, eight extracts (obtained from *Brachybacterium*, *Brevibacterium*, *Microbacterium*, *Rhodococcus*, and *Streptomyces* isolates) showed significant activity against at least one of the tested cancer cell lines (HepG2 and T-47D). Furthermore, 15 actinobacterial extracts showed anti-inflammatory potential in the RAW 264.4 cell model assay, with no concomitant cytotoxic response. Dereplication and molecular networking analysis of the bioactive actinobacterial extracts showed the presence of some metabolites associated with known natural products, but one of the analyzed clusters did not show any match with the natural products described as responsible for these bioactivities. Overall, we were able to recover taxonomically diverse actinobacteria with different bioactivities from the studied deep-sea samples. The conjugation of culture-dependent and -independent methods allows a better understanding of the actinobacterial diversity of deep-sea environments, which is important for the optimization of approaches to obtain novel chemically-rich isolates.

## 1. Introduction

The deep-sea is one of the most extensive habitat on earth, harboring highly diverse bacterial communities (Paulus, 2021). Deep-sea sediments are characterized by a variety of geophysical parameters that affect the associated microbial life. To face deep-sea conditions, microorganisms had to develop adaptation strategies, including the evolution of unique metabolic processes, to survive in this, often extreme, environment (Kamjam et al., 2017; Kerou et al., 2021). Due to the fact that the deep-sea is very vast and of difficult access, their bacterial communities remain broadly unfamiliar (Chen et al., 2016; Roman et al., 2019). Among the prokaryotic groups present in marine sediments, actinobacteria are often reported as one of the most predominant (Pathom-Aree et al., 2006; Chen et al., 2016; Jose and Jha, 2017). These microorganisms have also been shown to have high diversity in deep-sea sediments, of which a great share is predicted to be associated to novel species and genera (Bull et al., 2005).

Marine actinobacteria have revealed to have unique metabolic and physiological capabilities, translating into a high potential for the biosynthesis of novel metabolites (Yang and Song, 2018; Yang et al., 2020). Compounds synthesized by these microorganisms were shown to exhibit various bioactivities, such as antibacterial (Bull et al., 2005), antifungal (Qi et al., 2019), anticancer (Silva et al., 2020), antioxidant (Dholakiya et al., 2017; Kamjam et al., 2017), among others.

Until now, the diversity of actinobacteria associated with marine sediments, including deep-sea sediments, has been mostly investigated by culture-dependent methods. Limited efforts have been spent on the study of actinobacterial community composition of such habitats by conjointly employing culture-independent methods, such as high-throughput sequencing approaches (Chen et al., 2016; Jose and Jha, 2017), which is essential to accurately elucidate the diversity of these microorganisms. Previous culture-independent studies of deep-sea sediments from unexplored regions, such as the Arctic, have already demonstrated a prevalence of actinobacteria in these samples (Fang et al., 2019), however few studies have focused on the concomitant actinobacterial isolation and analysis of their bioactive potential. On the other hand, the actinobacterial diversity associated with deep-sea sediments of the Atlantic Macaronesia region is scarcely explored by culture independent-methods, although actinobacteria isolated from deep-sea sediments have already shown interesting bioactive potential (Chen et al., 2016; Prieto-Davó et al., 2016; Siro et al., 2023).

In this context, we combined culture-dependent and -independent methods to investigate the actinobacterial diversity of deep-sea sediments from two underexplored regions: the Macaronesia region of the Atlantic Ocean, within the Portuguese Continental Shelf, which includes, among others, the archipelagos of Azores and Madeira, and the Mohn’s Treasure region, an inactive sulfide mound in the Arctic Mid-Ocean Ridge. In addition, the actinobacteria isolated from these samples were investigated in terms of their bioactivity, by screening their antimicrobial, anticancer and anti-inflammatory potential. To the best of our knowledge, this is the first study of actinobacteria diversity of deep-sea sediments from two distinct oceanic regions, coupling culture-dependent and -independent approaches with investigation of the respective bioactive potential.

## 2. Materials and methods

### 2.1. Sampling

Deep-sea sediment samples were obtained from two geographical locations, Atlantic and Arctic, and were acquired from the upper layers of different bathyal zones (Table 1) using a remotely operated vehicle (ROV). The Atlantic samples were obtained from two regions of the Portuguese Continental Shelf (Azores and Madeira). For the Azores, samples were collected between 1,072 and 1,167 m depth, in September 2016, during the oceanographic campaign EMEPC\PEPC\Luso\2016 in the Mid-Atlantic Ridge, North Atlantic Ocean. Sampling was carried out by the ROV “LUSO” of the Portuguese Task Group for the Extension of the Continental Shelf (EMEPC), which collected three sediment samples (CMA_L1, CMA_L2, and CMA_L4) with a mini-corer (upper 5 cm depth) and one composite sediment through suction from different points on the sediment surface (CMA_L3). For Madeira, samples (M_E147 and M_MA3) were collected between 2,300 and 3,199 m depth, using a box-corer, during the mission SEDMAR 1/2017 carried out by the Portuguese Hydrographic Institute in June 2017. The Arctic samples were obtained from Mohn’ Treasure, an inactive sulfide mound on the Arctic Mid-Ocean Ridge that is covered by a thick layer of fine sediments (Ramirez-Llodra et al., 2020). Sampling was conducted in the framework of the MarMine campaign, in the summer of 2016. Six samples (A_136, A_78, A_79, A_80, A_81, and A_82) of the top sediment layer were collected with push corers using a Triton work-class ROV at depths between 2,682 and 2,826 m.

**Table 1:** Site description of the deep-sea sediment samples used in this study.

Site	Site description	Sample Code	Sample property	Depth (m)	Latitude	Longitude
Atlantic - Azores	Portuguese Continental Platform	CMA_L1	Sediment corer	1,167	33.9175	−37.5053
		CMA_L2	Sediment corer	1,072	33.9230	−37.5103
		CMA_L3	Composite sediment	1,065 to 1,073	33.92297 start 33.91748 end	−37.5103 start −37.5053 end
		CMA_L4	Sediment corer	1,167	33.9175	−37.5054
Atlantic - Madeira	Portuguese Continental Platform	M_E147	Box-corer	3,199	30.2208	−16.1032
		M_MA3	Box-corer	2,300	32.5218	−16.9683
Arctic	Mohn’s Treasure Ridge	A_78	Layer 1–3 cm	2,682	81.56960	63.3248
		A_79	Layer 1–3 cm	2,682	81.56960	63.3248
		A_80	Layer 1–3 cm	2,822	81.56960	63.3248
		A_81	Layer 1–3 cm	2,683	81.56913	63.3277
		A_82	Layer 10–15 cm	2,683	81.56915	63.3283
		A_136	Layer 0–1 cm	2,826	81.56505	63.3945

For each site, two sediment samples were collected and stored under different conditions. One was stored at −80°C for genomic analysis and the other was preserved with LifeGuard™ Soil Preservation Solution (MO BIO Laboratories; Carlsbad, CA, United States), under refrigerated conditions, for bioprospection of deep-sea actinobacteria.

### 2.2. DNA library preparation and amplicon sequencing

DNA of the sediment samples was extracted from 0.5 g of sediment using the Power Soil DNA Isolation Kit (MO BIO Laboratories; Carlsbad, CA, United States), following the manufacturer’s instructions. The extracted DNA samples were quantified and sent for sequencing at Genoinseq (Cantanhede, Portugal) company. The hypervariable V4-V5 regions of the 16S rRNA gene were amplified using the universal primers 515YF (5-GTGYCAGCMGCCGCGGTAA-3) and Y926R (5-CCGYCAATTYMTTTRAGTTT-3), developed by Parada et al. (2016). Pair-end sequencing with MiSeq® V3 chemistry was performed according to the manufacturer’s instructions (Illumina, San Diego, CA, United States). Raw reads extracted from Illumina MiSeq ® System were demultiplexed and pre-processed. Sequences were quality-filtered with PRINSEQ software and merged by using the AdapterRemoval v2.1.5 software (Schubert et al., 2016) with default parameters. Full description of the protocol is reported in (Bragança et al., 2019). Raw Illumina fastq files obtained in this study were deposited in the European Nucleotide Archive (ENA) database under the accession number PRJEB59507.

### 2.3. Bioinformatics analysis

Raw reads provided by the Genoinseq sequencing company were then processed in the present work by Cutadapt plugin for primer removal (Martin, 2011). Later, amplicon sequences were submitted to the open-source software Quantitative Insights into Microbial Ecology (QIIME 2; v.2019.7; Bolyen et al., 2019) for the upstream analysis. Dada2 algorithm within QIIME2 was used to merge paired-end reads (Callahan et al., 2016), denoise, dereplicate sequences, remove chimeras, and identify Amplicon Sequence Variants (ASVs) with default settings (Callahan et al., 2016), providing as output a ASV abundance table.

Taxonomy assignation of the ASV representative sequences was carried out against SILVA v 132 database (Quast et al., 2012) trained with a naïve Bayes classifier against the same the V4-V5 region of 16S rRNA gene target for sequencing. ASV assigned to non-target sequences (e.g., “Eukarya,” Choloroplasts,” and “Mitochondria”) and not classified at Kingdom level were removed and excluded from the analysis. Rarefaction curves were obtained through QIIME 2 core diversity metrics to exhibit the sequencing depth of samples. Taxonomy plots were created using the ggplot2´ package in R environment (version 3.2.2. Copyright 2015 The R Foundation for Statistical Computing).

### 2.4. Actinobacteria isolation and phylogenetic identification

For the isolation of actinobacteria from the deep-sea sediment samples, each sediment was initially shaken for 30 min at 150 rpm to homogenize the sample. One gram of each sediment sample was transferred to an eppendorf and suspended in 1 ml of sterile seawater. Pre-treatments were applied to all samples except for the Azores sediments (Table 2). For the Madeira samples the following pre-treatments were applied: (i) incubation in a water bath at 60°C for 30 min (wet heat; Terahara et al., 2013); (ii) incubation at 120°C for 60 min (dry heat; Bredholt et al., 2008) and (iii) incubation in a microwave at 120 W for 3 min (Bredholdt et al., 2007). The Arctic sediment samples were processed using both a wet heat pre-treatment and no pre-treatment. After applying the pre-treatment (s; when applicable), samples were vortexed at maximum speed for 5 min, to dissociate the bacteria from the sediments and transfer them to the sterile seawater, after which 10-fold dilutions (until 10^−4^) were prepared. An aliquot of 100 μl of each dilution was spread over the surface of different isolation media, according to the information indicated in Table 2. The media used were: Gauze’s Synthetic Medium No. 1 (per liter, with a ratio of seawater: deionized water of 70: 30): 20 g of soluble starch, 1 g of KNO_3_, 0.5 g of K_2_HPO_4_, 0.5 g of MgSO_4_.7H_2_O, 0.05 g of FeSO_4_.7H_2_0 and 17 g of agar; Starch-casein-nitrate agar (SNC; per liter, with a ratio of seawater: deionized water of 70: 30): 10 g of soluble starch, 0.3 g of casein, 2 g of K_2_HPO_4_, 2 g of KNO_3_, 2 g of NaCl, 0.05 g of MgSO_4_.7H_2_O, 0.02 g of CaCO_3_, 0.01 g of FeSO_4_.7H_2_0 and 17 g of agar; M1 agar (per liter of seawater): 10 g of soluble starch, 4 g of yeast extract, 2 g of peptone and 17 g of agar; Nutrient-poor sediment extract (NPS; per liter of seawater): 100 ml of marine sediment extract (obtained by washing 900 ml of sediments with 500 ml of seawater) and 17 g of agar; and Raffinose-Histidine (RH; per liter, with a ratio of seawater: deionized water of 70: 30): 10 g of raffinose, 1 g of L-histidine, 1 g of KH_2_PO_4_, 0.5 g of MgSO_4_.7H_2_O, 0.01 g of FeSO_4_.7H_2_0 and 17 g of agar.

**Table 2:** Pre-treatments and culture media used for the isolation of actinobacteria from the deep-sea sediments.

Site	Site description	Sample Code	Pre-treatments	Culture media
Atlantic - Azores	Portuguese Continental Platform	CMA_L1	Not applied	Gauze, SCN and RH
		CMA_L4		
		CMA_L3		
		CMA_L4		
Atlantic - Madeira	Portuguese Continental Platform	M_E147	(i) Wet heat(ii) Dry heat(iii) Microwave	SCN, NPS and M1
		M_MA3		
Arctic	Mohn’s Treasure Ridge	A_78	Not applied (i) Wet heat	SCN, NPS and M1
		A_79		
		A_80		
		A_81		
		A_82		
		A_136		

All media were supplemented with cycloheximide (50 mg/L), nystatin (50 mg/L) and nalidixic acid (50 mg/L) to reduce the growth of fungi and Gram-negative bacteria. The plates were incubated for a period of up to 6 months at 28°C. Along the incubation period, plates were regularly inspected and colonies with different morphological characteristics were picked and streaked on new agar plates until obtainment of pure colonies.

Biomass for cryopreservation and DNA extraction was obtained by growing each isolate in the corresponding isolation liquid medium, with the exception of those obtained from the NPS medium that were grown in marine broth (Laboratorios Conda, Madrid, Spain). Each pure isolate was cryopreserved at −80°C in 30% (v/v) glycerol (Passari et al., 2018). For DNA extraction, 1 ml of liquid culture was centrifuged for 5 min at 7,000 *g* and the pellet was stored at −20°C. The E.Z.N.A. Bacterial DNA kit (Omega Bio-Tek, GA, United States) was used for DNA extraction, following the instructions of the manufacturer, with a few modification steps described by Albuquerque et al. (2021). 16S rRNA gene was amplified by Polymerase Chain Reaction (PCR) using the universal primers 1492R (5’-GGTTACCTTGTTACGACTT-3′) and 27F (5’-GAGTTTGATCCTGGCTCAG-3′) as described by Ribeiro et al. (2020). Purification and sequencing of the amplified fragments were performed at GenCore, i3S (Instituto de Investigação e Inovação em Saúde, Portugal). The sequences were analyzed using the Geneious software package (version 11.1.4) and the resulting consensus sequences were compared against the databases: 16S ribosomal RNA (Bacteria and Archaea), nucleotide collection (nr/nt), both from NCBI BLAST, using the blastn algorithm,^1^ and the 16S-based ID tool from EZBioCloud.^2^ The 16S rRNA gene sequences of the isolated actinobacterial strains were deposited in GenBank® (NCBI, Maryland, United States) with the accession numbers indicated in Supplementary Table S1.

### 2.5. Preparation of actinobacterial crude extracts

For the preparation of crude extracts, the actinobacterial isolates cryopreserved at −80°C were first grown in the respective agar medium in which they were isolated. A loopful of each isolate was used to prepare a pre-inoculum in falcon tubes containing 5 ml of the respective culture medium (with the same composition of the isolation medium, but without the addition of antibiotics), which was after used to inoculate 100 ml Erlenmeyer flasks containing 30 ml of culture medium. The flasks were incubated at 28°C, 100 rpm, in the dark for 3–5 days (depending on their growth rate) in a rotatory incubator (Model 210, Comecta SA, Barcelona, Spain), after which 0.5 g of Amberlite® XAD16N resin (Sigma-Aldrich, MO, United States) was added to the cultures (to adsorb compounds produced by the actinobacteria and eventually released to the culture medium) and left to incubate for an additional period of 2–3 days (Ribeiro et al., 2020). The biomass and resin were centrifuged at 3,600 *g* for 10 min, washed twice with dH2O and freeze- dried. The freeze-dried biomass and resin were then extracted with a 1:1 (v/v) mixture of acetone and methanol. The biomass was initially immersed in the mixture (30 ml) for 30 min with constant agitation, then centrifuged (4,500 *g* for 10 min) and the liquid phase was collected in round bottom flasks after passage through a Whatman No1 filter paper. The process was repeated twice, and the extract was dried in a rotary evaporator and transferred to a vial. The organic extract was then dissolved in DMSO (DMSO ≥99.9%, Sigma-Aldrich, MO, United States) to obtain stock solutions with final concentrations of 10 and 1 mg/ml.

### 2.6. Bioactivity assays

#### 2.6.1. Antimicrobial bioactivity screening

All the actinobacterial extracts were tested for antimicrobial activity through the agar-based disk diffusion method. In this assay, five reference microorganisms were tested: two Gram-positive bacteria - *Bacillus subtilis* (ATCC 6633) and *Staphylococcus aureus* (ATCC 29213); two Gram-negative bacteria - *Escherichia coli* (ATCC 25922), and *Salmonella typhimurium* (ATCC 25241); and one Yeast - *Candida albicans* (ATCC 10231). The bacterial strains were grown in Mueller-Hinton agar (MH; Liofilchem, Roseto d. Abruzzi, Italy) and the yeast strain was grown in Sabouraud dextrose agar (SD; Liofilchem, Roseto d. Abruzzi, Italy).

For each strain, an inoculum was prepared in the respective culture broth and the turbidity of the cultures was adjusted to 0.5 McFarland standard scale (OD_625_ = 0.08–0.13). These cultures were used to seed MH (for the bacterial strains) or SD (for the yeast strain) agar plates by uniformly streaking the plates with a swab. Sterile blank paper discs (6 mm in diameter; Oxoid Limited, Hampshire, United Kingdom) were placed on the top of the inoculated plates and loaded with 15 μl of each actinobacterial extract at a concentration of 1.0 mg/ml. For the negative controls, 15 μl of molecular biology grade dimethyl sulfoxide (DMSO; Scharlab, Barcelona, Spain) were loaded on the blank paper discs, while for the positive controls the paper disks were loaded with 15 μl of enrofloxacin (1.0 mg/ml; Sigma-Aldrich, MO, United States) for the bacterial strains and 15 μl of nystatin (1.0 mg/ml; Sigma-Aldrich, MO, United States) for the yeast. Agar plates were incubated at 37°C for 24 h, and the diameter of halos corresponding to a zone of growth inhibition was measured. Each extract was tested in two independent assays.

Minimum inhibitory concentration (MIC) of the bioactive crude extracts was also determined using *Candida albicans* as inoculum. For each extract, solutions were prepared at 1000 mg/ml in SD broth. Two-fold dilutions in the same culture medium were performed to obtain extracts with concentrations ranging from 1,000 to 0.5 mg/ml. In 96-well plates, 50 μl of *C. albicans* inoculum (diluted 1:100) and 50 μl of each extract dilution were added to each well. The MIC values were established by spectrophotometry at 625 nm (model V-1200, VWR, PA, United States), after 18 h of incubation at 37°C. The controls consisted of: (i) negative control - 100 μl of broth medium; (ii) positive control - 50 μl of yeast inoculum and 50 μl of broth medium; and (iii) solvent control - 50 μl of yeast inoculum and 50 μl of DMSO (DMSO ≥99.9%, Sigma-Aldrich, MO, United States). Assays were performed in triplicate and in two independent experiments.

#### 2.6.2. Anticancer bioactivity screening

The screening of anticancer activity in the actinobacterial extracts was performed in two cancer cell lines: breast ductal carcinoma (T-47D) and liver hepatocellular carcinoma (HepG2), both from Sigma-Aldrich (St. Louis, MO, United States), as well as in a non-cancer cell line hCMEC/D3 (human brain capillary endothelial cells), kindly donated by Dr. P. O. Courad (INSERM, France), which was used to access general toxicity. Dubelco’s modified eagle medium (DMEM; Gibco, Thermo Fischer Scientific, Waltham, MA, United States) supplemented with 10% (v/v) fetal bovine serum (Biochrom, Berlin, Germany), 1% (v/v) penicillin/streptomycin (Biochrom, Berlin, Germany) at 100 IU/ml and 10 mg/ml, respectively, and 0.1% (v/v) amphotericin (GE Healthcare, Little Chafont, UK) was used for the growth and maintenance of the cell lines. The cells were incubated at 37°C in a humidified atmosphere containing 5% of CO_2_ (Biosystem w/o Controller, Cimarec., Thermo Fischer Scientific, MA, United States). Cellular viability was evaluated using the MTT (3-(4,5-dimethylthiazol-2-yl)-2,5-diphenyltetrazolium bromide) colorimetric assay. The cells were seeded in 96-well plates at a density of 6.6×10^4^ cells/ml. After 24 h, the cells were exposed to the actinobacterial extracts at a final concentration of 15 μg/ml, during 48 h. After this period, the cells were incubated with MTT at a final concentration of 0.2 mg/ml (Sigma-Aldrich, MO, United States) for 3–4 h at 37°C. The medium was then removed and 100 μl of DMSO were added at each well to dissolve formazan crystals. Absorbance was read at 570 nm on a multi-detection microplate reader (Synergy HT, Biotek, Bart Frederick Shahr, Germany). Staurosporine (at a final concentration of 15 μg/ml) and 0.5% DMSO (same concentration as actinobacterial extracts) were used as a positive and solvent controls, respectively. Cellular viability was calculated as a percentage relative to the solvent control. The extracts were tested in triplicate in each two independent assays (*n* = 6).

#### 2.6.3. Anti-inflammatory bioactivity screening

The mouse macrophage cell line (RAW264.7) was used for the study of anti-inflammatory activity. This cell line was kept at 37°C in a 5% CO_2_ incubator and maintained in Dulbecco’s Modified Eagle’s Medium (DMEM) supplemented with 10% (v/v) fetal bovine serum (Biochrom, Berlin, Germany), 1% (v/v) penicillin/streptomycin (Biochrom, Berlin, Germany) at 100 IU/ml and 10 mg/ml, respectively, and 0.1% (v/v) amphotericin (GE Healthcare, Little Chafont, UK). For the seeding of the cells, confluent macrophage cells (3.5 × 10^5^ cells) were added to 96 well plates and incubated at 37°C for 24 h. In the next day, the medium was renewed, and the cells were exposed to the actinobacterial extracts at a final concentration of 15 μg/ml, for 24 h. In the anti-inflammatory assay, the cells were pre-treated with LPS (lipopolysaccharide, Sigma-Aldrich) at a final concentration of 200 μg/ml, while in the pro-inflammation assay no LPS was added to the wells. Solvent control consisted of 0.5% DMSO and the positive control of LPS at 200 mg/ml. Inflammation was analyzed by Nitric Oxide (NO) level using the Griess reaction. Seventy-five μl of the supernatant of the cell culture were transferred to a new 96 well plate and NO was quantified by adding 75 μl Griess reagent (1% Sulfanilamide (w/v) in 2% phosphoric acid, 0.1% N-(1-Naphthyl) ethylenediamine dihydrochloride). The plates were incubated for 15 min in the dark and the level of NO was determined by measuring the absorbance at 562 nm. Additionally, the MTT assay was conducted to determine the cell viability in each condition by adding MTT to the original 96 well plates at a final concentration of 0.5 mg/ml (Sigma-Aldrich, MO, United States) and incubating the plates for 45 min at 37°C. The medium was then removed and 100 μl of DMSO were added at each well, after which the absorbance was read at 510 nm (Synergy HTX, Biotek, Winooski, VT, United States).

#### 2.6.4. Statistical analysis

Results from the anticancer and anti-inflammatory assays were statistically analyzed by comparing the results obtained in the extracts and in the solvent control. Firstly, the Kolmogorov Smirnov test was used to verify normality distribution of each data, and Bartlett’s test for equal variances. For parametric data, one-way ANOVA followed by Dunnett’s *post hoc* test was applied. For nonparametric data, the Kruskal-Wallis test was used followed by Dunn’s multiple comparison test. The significance level established for all tests was at *p* < 0.05.

### 2.7. Dereplication and molecular networking analysis

The actinobacterial extracts that showed bioactivity in the performed bioassays were selected for mass spectrometry (MS)-based dereplication and molecular networking analysis. Initially, the bioactive extracts were resuspended in methanol (at a final concentration of 2 mg/ml) and analyzed by liquid chromatography-high resolution electrospray ionization tandem mass spectrometry (LC-HRESIMS/MS) to identify crude extracts with potentially new bioactive compounds. This analysis was performed on a Dionex Ultimate 3,000 HPLC coupled to a qExactive focus mass spectrometer controlled by XCalibur 4.1 software (Thermo Fisher Scientific, United States). The chromatographic step was conducted under the same conditions as described by Ribeiro et al. (2020). The resulting raw data from MS-based dereplication was converted to the mzML format and submitted to the Global Natural Products Social Molecular Networking (GNPS) platform to analyze if the extracts showed significant hits that could explain the activities observed (Wang et al., 2016). Three dereplication analyses were used: Insilico Peptidic Natural Product Dereplicator, Dereplicator VarQuest and Dereplicator+, with default parameters, excluding ion mass precursor tolerance and fragment ion mass tolerance (set to 0.005 Da). In addition, a molecular network was constructed using the GNPS data analysis workflow, applying default parameters (except precursor ion mass tolerance and fragment ion mass tolerance which were set to 0.02 Da). The molecular network was loaded into Cytoscape v3.8.2 to visualize the sets of spectra from related molecules and the distribution of m/z clusters among actinobacterial extracts. For this analysis we only focused on unique single-strain m/z clusters and manually checked the corresponding LC-HRESIMS chromatograms to determine the mass of each target metabolite. The deduced masses from m/z values for major compounds were submitted to two databases of natural products, the Dictionary of NP (version 27.1, CRC Press, Abingdon, UK) and the NP atlas database (van Santen et al., 2022), to check if they corresponded to compounds previously reported from Actinobacteria.

## 3. Results

### 3.1. 16S rDNA metabarcoding analysis of deep-sea sediments

In order to survey the abundance and diversity of actinobacteria in the different deep-sea sediments, a culture-independent approach based on the high-throughput sequencing of 16S rRNA gene amplicons was implemented. The sequencing effort produced a total of 769,127 sequences of V4-V5 16S rRNA gene with an average sequencing depth of ~64 k reads per sample. Rarefaction curves of the 12 sequenced samples reached a horizontal asymptote, indicating that an adequate sequencing depth was achieved for all samples (Supplementary Figure S1).

**Figure 1:**
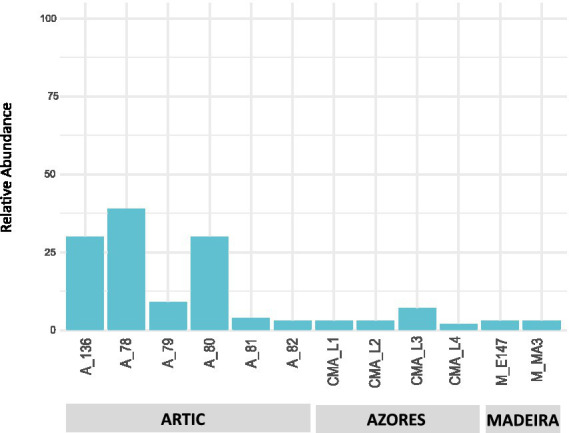
Taxonomic diversity and relative abundance at the phylum level of bacteria from the deep-sea sediment samples. Unassigned sequences and phyla comprising <2% of the total number of ASVs were classified as “Other.”

**Figure 2:**
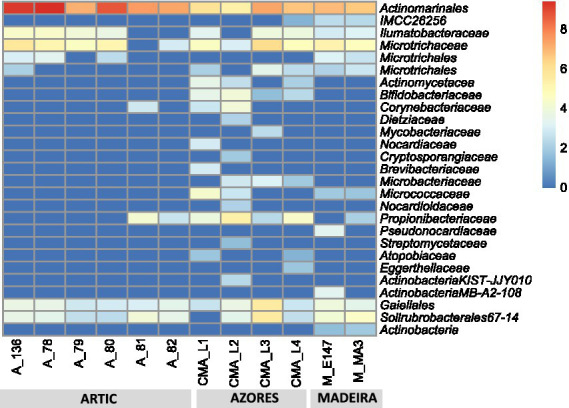
Heatmap with log transformation of the ASV abundance of Actinobacteria at order and family taxonomic levels in all deep-sea samples.

**Figure 3:**
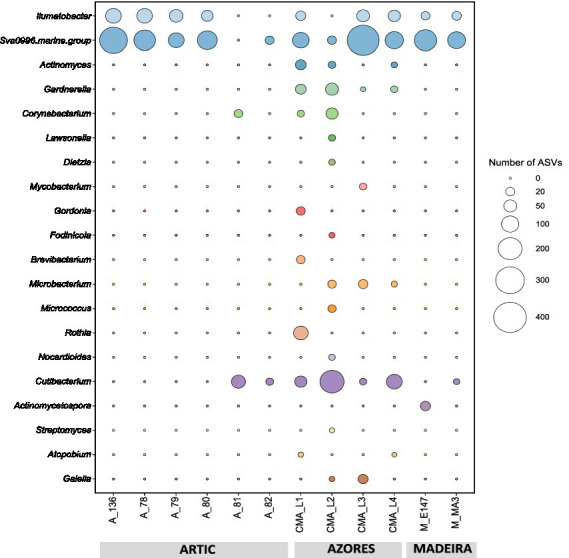
Absolute abundances (as number of ASVs) of the actinobacterial genera found across the deep-sea samples.

Results showed that the Actinobacteria phylum was particularly abundant in the Arctic deep-sea sediments, in some cases ranking as the first or second most dominant phylum, ranging from 3 to 31%, but showed a lower representation in the Madeira and Azores samples, with relative abundances never exceeding 7% (Figure 1). A deeper look into the actinobacterial community of these samples reveals interesting features. On the one hand, the high dominance of the Actinobacteria phylum in the Arctic deep-sea sediments was apparently driven by the high abundances of only a handful of actinobacterial taxa, particularly those accommodated in the *Actinomarinales* order as well as in the *Ilumatobactereaceae* and *Microtrichaceae* actinobacterial families (Figure 2). The most prominent actinobacterial genera accommodated in these taxa was *Illumatobacter* and those affiliated with the *Sva0996 marine group* (Figure 3). On the other hand, the Madeira and Azores deep-sea sediments revealed a higher diversity of actinobacterial families (13 and 22, respectively; Figure 3), despite the lower overall prevalence of the Actinobacteria phylum in these samples. At the genus level, however, the Azores deep-sea samples stood out, as they exhibited a much higher number of different actinobacterial genera (19 in total) than the Madeira samples, which showcased a total of only 4 different actinobacterial genera (Figure 3).

### 3.2. Actinobacteria isolated from the deep-sea sediments

A total of 44 actinobacterial isolates were obtained from the 12 deep-sea sediment samples analyzed (Supplementary Table S1). These isolates were distributed by nine genera: *Actinotalea*, *Brachybacterium*, *Brevibacterium*, *Dietzia*, *Leucobacter*, *Microbacterium*, *Micrococcus*, *Rhodococcus*, and *Streptomyces*. The largest fraction was associated with *Brachybacterium* (11 isolates), *Microbacterium* (11 isolates), and *Brevibacterium* and (10 isolates) genera. Regarding the distribution of the isolates per region, actinobacterial strains isolated from the Arctic and Madeira samples were mostly associated with the *Brachybacterium* and *Brevibacterium* genera, while isolates from Azores sediments were predominantly affiliated with the genus *Microbacterium*. Among the less abundant genera, *Actinotalea* and *Dietzia* were only retrieved from the Arctic sediments, *Rhodococcus* was obtained only from the Azorean sediments and *Leucobacter* was solely isolated from the Madeira sediments.

The pre-treatments applied to the samples from the Arctic and Madeira regions did not produce a significant effect on the number of actinobacteria recovered from each sample. However, the genera *Actinotalea* and *Dietzia* were only retrieved from the Arctic samples subjected to the wet heat pre-treatment, while this same pre-treatment allowed the recovery of the genera *Streptomyces* and *Leucobacter* in the samples from Madeira. No actinobacterial growth was obtained in the Madeira samples exposed to the pre-treatment dry heat, indicating that this treatment was overly selective.

Taxonomic analysis of some of the retrieved isolates indicate that they may constitute new species, in the light of the similarity threshold for a new species of 98.7% (Supplementary Table S1; Stackebrandt, 2006). Of these, *Streptomyces* strain MA3_2.13 was already proven to be a new species (Albuquerque et al., 2021).

### 3.3. Bioactivity screening of the actinobacterial extracts

The crude extracts from all deep-sea actinobacterial isolates were tested for their antimicrobial, anticancer and anti-inflammatory activities. Two actinobacterial crude extracts, obtained from the *Streptomyces* strains MA3_2.14 and 82_2.13, isolated from the Madeira and Arctic regions, respectively, exhibited antimicrobial activity against *C. albicans* (Table 3). The Actinobacteria isolated from the Azores sediments did not show antibacterial activity against any of the pathogenic strains used.

**Table 3:** Actinobacterial crude extracts with antimicrobial activity. MIC: Minimal inhibitory concentration.

Site	Strain	Closest taxonomic identification	Disk diffusion method	
			*C. albicans* (inhibition halo mm)	MIC (μg/ml)
Madeira	MA3 2.14	*Streptomyces* sp.	21	7.81
Arctic	82_2.13	*Streptomyces* sp.	16	15.62

**Figure 4:**
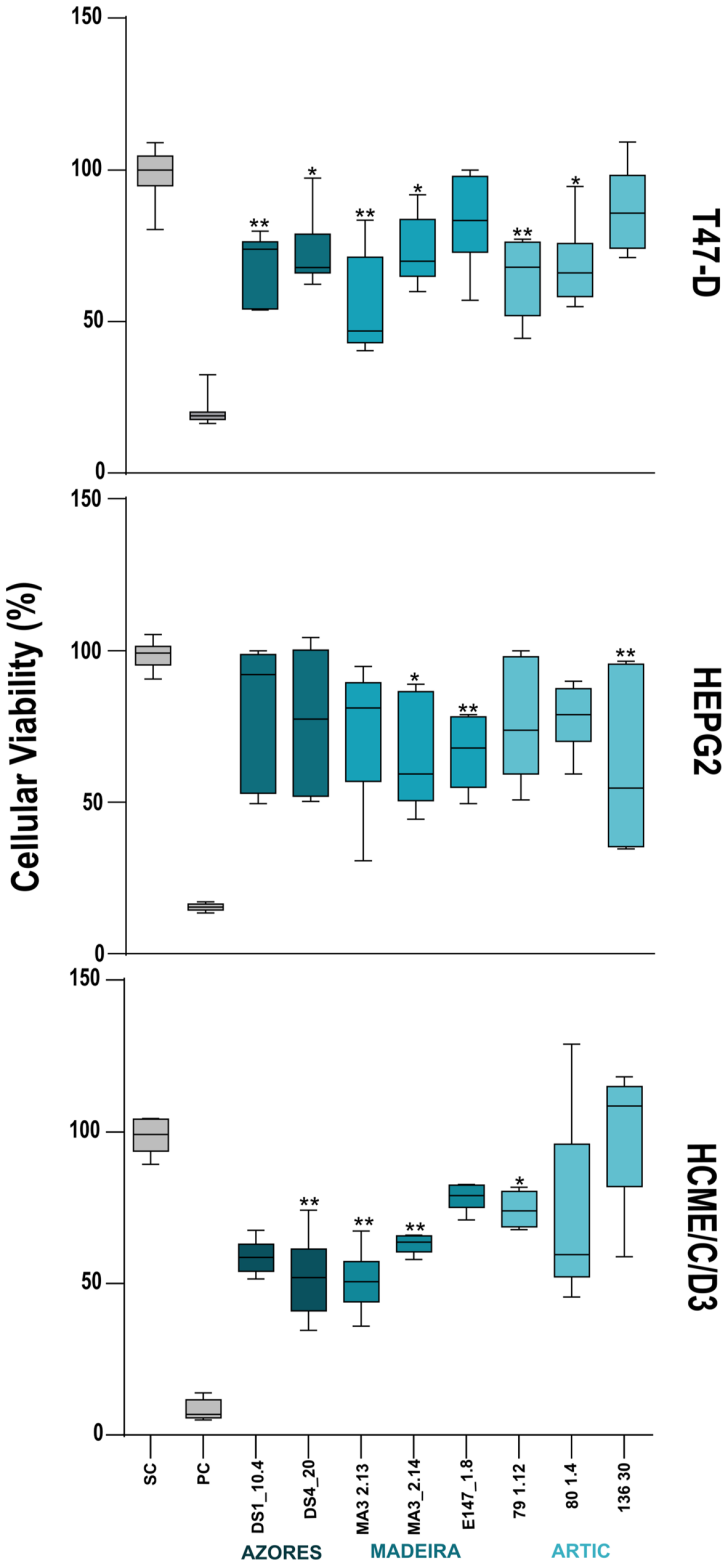
Cytotoxic activity of the deep-sea actinobacterial crude extracts tested at 15 μg/ml. The effects on the viability in the two cancer cell lines: breast ductal carcinoma (T-47D) and liver hepatocellular carcinoma (HepG2), as well as in the non-cancer cell line hCMEC/D3 (human brain capillary endothelial cells) are shown after 48 h of exposure. Only the extracts with statistically significant differences are indicated on the graphs. PC and SC mean positive and solvent controls, respectively. Values are represented as interval displaying the data distribution through their quartiles with 5–95% confidence from two independent assays conducted in triplicate and line within the box-whisker represents the median. Significant differences compared to the solvent control are annotated with asterisks in the graphs (*p < 0.05; **p < 0.01; ***p < 0.001; ****p < 0.0001).

Eight actinobacterial crude extracts demonstrated significant cytotoxic activity against one or both cancer cell lines tested (HepG2 and T-47D). Actinobacterial strains belonging to the genera *Microbacterium* (DS1_10.4), *Rhodococcus* (DS4_20), *Streptomyces* (MA3_2.13 and MA3_2.14), *Brevibacterium* (79_1.12), and *Brachybacterium* (80_1.4) reduced the cellular viability of the T47-D line in more than 30% (Figure 4). The extracts from the strains *Brevibacterium* 136.30 and *Brachybacterium* E147_1.8 reduced the viability of the HepG2 cancer line in more than 35%. Curiously, crude extracts from strains DS1_10.4, 80 1.4, 136.30 and E147_1.8 showed activity only in cancer cells lines and not in the non-tumor cell line hCMEC/D3 (Figure 4). On the other hand, the extracts DS4_20, MA3 2.13 and 79_1.12 showed a generalized toxicity, reducing the viability of the hCMEC/D3 cell line by more than 30%. The extract MA3_2.14 was the only one that showed activity in the three cell lines tested. Regarding to the described cytotoxicity profile, the extract E147_1.8 seems the most promising, reducing by 30% specifically the HepG2 cells, but not T47D and the non-carcinogenic cells.

**Figure 5:**
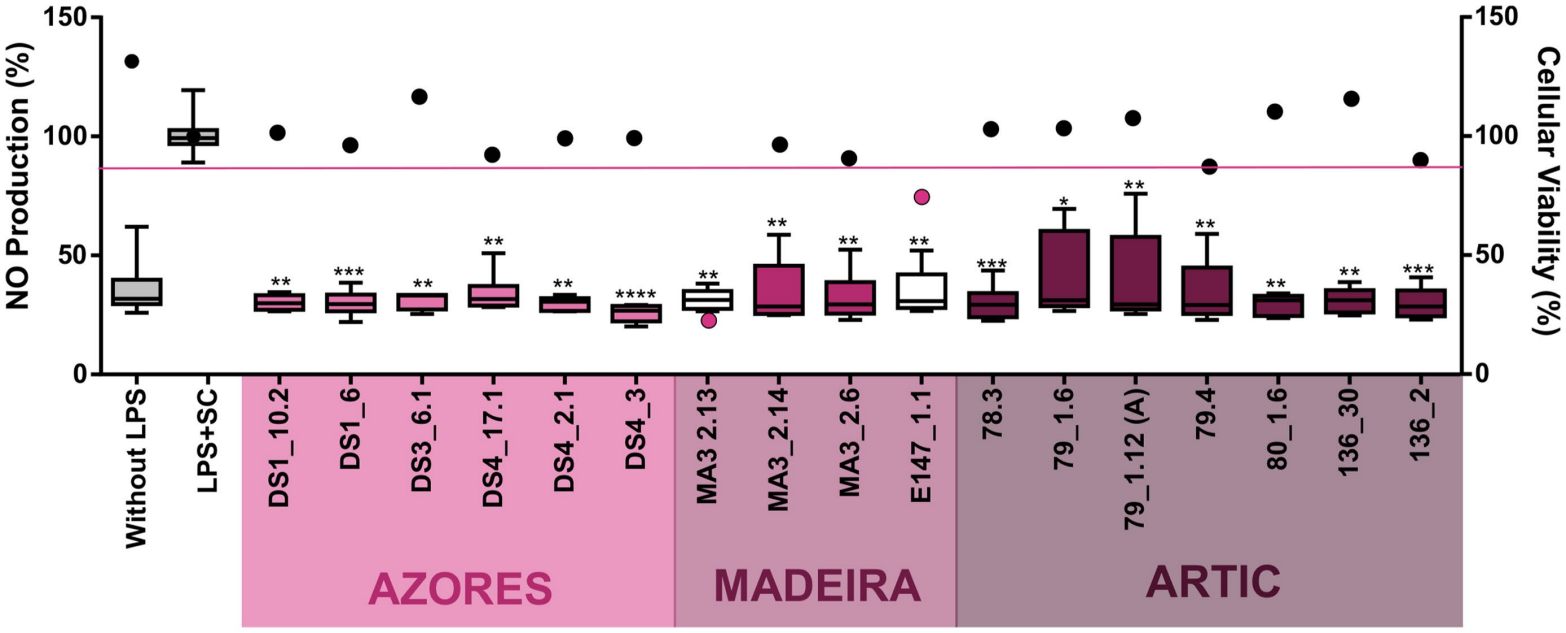
Anti-inflammatory activity of the deep-sea actinobacterial crude extracts (15 μg/ml) after inflammation induced by LPS exposure (200 μg/ml). The inflammatory effect on the RAW 264.4 cell line as measured by NO production (box-whisker graph) and the effect on the viability of the same cellular line (plot graph) are shown after 48 h of exposure. Only the extracts with statistically significant differences are indicated in the graphs. All plot values represented below the pink line indicate that the extracts significantly reduce cell line viability in addition to having anti-inflammatory activity. Without LPS and LPS + SC denote positive and solvent with lipopolysaccharide controls, respectively. Values are demonstrated as interval displaying the data distribution through their quartiles with 5–95% confidence from two independent assays conducted in triplicate and line within the box-whisker represents the median. Significant differences compared to the solvent control are annotated with asterisks in the graphs (*p < 0.05; **p < 0.01; ***p < 0.001; ****p < 0.0001).

Regarding anti-inflammatory activity, 17 extracts decreased significantly the inflammatory response quantified by NO production. However, two of these extracts also reduced the viability of the macrophage cell line RAW264.7, indicating that the reduction of NO production in the presence of these extracts is a result of the decrease of cellular viability rather than of an anti-inflammatory effect (Figure 5). Fifteen actinobacterial extracts, derived from the *Brachybacterium* strains 78.3, 79_1.12A, 79_1.6 and 79.4, the *Brevibacterium* strains 80_1.6 and 136.30, the *Microbacterium* strains DS1_1.6, DS1_10.2, DS3_6.1, DS4_17.1 and DS4_2.1, the *Rhodococcus* strain DS4_3, the *Dietzia* stain 136.2, the *Leucobacter* strain MA3_2.6, and the *Streptomyces* strain MA3_2.14 had the ability to reduce the inflammatory response by more than 60%, without showing associated cytotoxicity (Figure 5). In terms of geographic location, these Actinobacteria were isolated from the three regions targeted in this work. Importantly, all the extracts with anti-inflammatory activity did not show pro-inflammatory properties. In addition, it should be emphasized that the extract obtained from the *Brevibacterium* strain 136.30 demonstrated both anti-inflammatory (70% reduction) and anticancer (33% inhibition of viability of the HepG2 cancer line) activities, while no activity was observed against the non-cancer cell line (hCMEC/D3). On the other hand, the *Streptomyces* strain MA3_2.14 showed a cytotoxic activity against all cell lines tested (including cancer and non-cancer cell lines) and also reduced inflammation without affecting the viability of the macrophage cell line (Figures 4, 5).

### 3.4. Dereplication and molecular networking analysis

The metabolic profile of the actinobacterial crude extracts that showed bioactivity was studied to understand if these bioactivities could be a result of known or new secondary metabolites. A dereplication analysis was performed on 22 extracts using Insilico Peptidic Natural Products tools available in the GNPS Platform. For analysis of the results, a threshold *p*-value ≤ 10^−10^ for DEREPLICATOR (Mohimani et al., 2017) and DEREPLICATOR VarQuest (Gurevich et al., 2018), and a very strict score value of 15 on DEREPLICATOR+ were selected to reduce false matches (Mohimani et al., 2018). After this, all matches for compounds not produced by Actinobacteria were discarded.

For the DEREPLICATOR, the results showed the presence of the peptide Surugamide A in the extract of the *Streptomyces* strain 82_2.13, obtained from Arctic sediments, and Surugamides B and D in the extract of the *Streptomyces* strain MA3_2.14, derived from Madeira sediments. When using DEREPLICATOR with the VarQuest algorithm, 23 matches were obtained for only eight actinobacterial extracts, five derived from strains of the Arctic region (*Brachybacterium* sp. strain 78.3 and strain 79.4, *Brevibacterium* sp. strain 80_1.6, *Dietizia* sp. strain 136_2 and *Streptomyces* sp. strain 82_2.13), two from strains isolated from the Azores archipelago (*Microbacterium* sp. strains DS1_10.4 and DS3_6.1) and one from a *Streptomyces* sp. strain MA3 2.14 from Madeira. This analysis allowed the annotation of analogues of anticancer compounds and lipopeptides antibiotics. DEREPLICATOR+ *in silico* tool allowed the annotation of different secondary metabolites in 13 actinobacterial extracts derived from strains from all the regions studied. However, it is important to mention that extracts belonging to *Brachybacterium* sp. strains 80_1.4 and 79_1.6, both from the Arctic region, *Microbacterium* sp. strain DS1_1.6, DS1_10.2, DS4_17.1 and DS4_2.1 obtained from the Azores region, and *Leucobacter* sp. strain MA3_2.6 from the Madeira region, did not show matches with any secondary metabolite that could explain the anticancer and anti-inflammatory activities observed in these extracts (Supplementary Table S2).

To complement these data, a molecular network was additionally constructed using GNPS. We found a total of 16 clusters that are shared by two or more actinobacterial extracts and nine belonging only to a single extract, which we specified as the main criterion selected to increase the possibility that a cluster corresponds to an unknown compound and, therefore, to undergo more dereplication analysis using more comprehensive, non-MS/MS based databases. Concerning the single-strain clusters, four were discovered in the extract obtained from *Streptomyces* sp. MA3_2.13 and two others in the extract from *Brachybacterium* sp. E147_1.18, both derived from the Madeira region. Three additional single-strain clusters were found in the extract of *Microbacterium* sp. DS3_6.1 isolated from Azores sediments.

**Figure 6:**
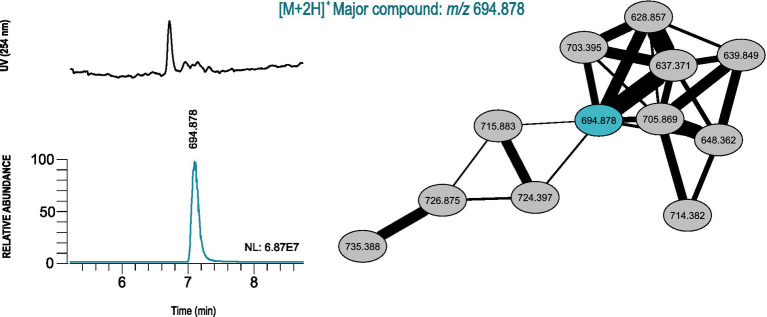
GNPS-based LC-HRESIMS/MS molecular networking analysis of the actinobacterial crude extract from *Microbacterium* sp. DS3_6.1, retrieved from the Arctic region, which probably contains news natural products. Unique molecular cluster represented by connected ellipses and marked with the corresponding m/z value. The major compound is estimated from the UV chromatogram and EICs, and shown in a different color from the remaining cluster nodes. The NL (normalization level) value corresponds to the base peak intensity of the m/z value when compared with the mass composition of the actinobacterial crude extract.

The extracted ion chromatogram (EIC) from each m/z value was analyzed for all single-strain clusters, to understand the relative abundance and look for the presence of common adduct ions (such as hydrogen, sodium and ammonia). After this analysis, only four clusters were selected and the most abundant m/z values in each cluster were used to calculate an accurate mass to be used as query in the Dictionary of NP (version 27.1) and NP atlas databases. For three clusters (major compound m/z 297.17, m/z 387.180 and m/z 543.369), hits from compounds produced by actinobacteria were obtained that correspond to the exact masses of known natural products (methyl 2-(3,5-dimethyl-2-oxocyclohexyl)tetrahydro-6-oxo-2H-pyran-4-acetate, Furaquinocin H and Milbemycin β2, respectively). For the cluster from the extract of *Microbacterium* sp. DS3_6.1 (major compound m/z 694.878), no hits were found in the dereplication, indicating that this cluster may be a new metabolite. The chromatographic peak corresponding to the most abundant m/z value in the cluster revealed that it will probably be produced in sufficient quantities, under the employed culture conditions, to allow for its isolation after scaling-up (Figure 6; Supplementary Figure S2).

## 4. Discussion

The development of new technology, mainly involving unmanned underwater platforms, such as remote operated vehicles, has allowed a rapid progress in deep-sea research that is accompanied by an increase for the exploration and exploitation of deep-sea resources (Ramirez-Llodra et al., 2011). However, our capability to develop robust management measures that are essential to balance resource use and ecosystem conservation, is hindered by our limited understanding of the composition, diversity and functioning of many deep-sea ecosystems (Howell et al., 2021). In this study, we explored the biodiversity of actinobacteria associated with deep-sea sediments from the Azores and Madeira regions, within the limits of the Portuguese Continental Shelf, and the Arctic Mid-Ocean Ridge, through culture-dependent and -independent methods. In parallel to this, we also studied their bioactivities and potential to produce new natural products.

The deep-sea sediments analyzed in our study showed that the Actinobacteria phylum has the highest representation in the Arctic deep-sea sediments, which is in line with other deep-sea biodiversity studies performed in this region (Hoffmann et al., 2017; Fang et al., 2019), including deep-sea sediments from the Mohn’s Ridge, from where our samples were collected (Lysnes et al., 2004). As for the deep-sea sediments from the Madeira archipelago, where the lowest proportion of the Actinobacteria phylum was observed, no studies on the prokaryotic diversity of deep-sea sediments from this region have been reported.

The abundance and diversity of actinobacteria in the deep-sea sediments seemed to be negatively correlated. For instance, most of the dominance of the Actinobacteria phylum in the Arctic deep-sea samples was driven by the predominance of *Actinomarinales*, *Ilumatobacteraceae*, and *Microtrichaceae*, which represented on average 97% of the Actinobacteria phylum, while in the Azores and Madeira deep-sea sediments it was noted a higher diversity of actinobacterial taxa (including the dominance of *Actinomarinales*), despite their lower overall relative abundances in the prokaryotic microbiomes of these sediments. Still, the high prevalence of *Actinomarinales* in the deep-sea sediments across the different regions studied is likely correlated with the cosmopolitan distribution of this taxon across the marine environment (Ghai et al., 2013), having also been observed in deep-sea sediments from São Paulo Plateau, in the Atlantic Ocean (Queiroz et al., 2020). Yet, this abundance-diversity dichotomy is less expressive at the genus level, probably due to a low resolution of taxonomic classifications achieved in this study. At this taxonomic level, *Illumatobacter* and *Sva0996 marine group* were the most abundant and more frequently detected actinobacterial genera across all samples analyzed, representing the major genera identified in the Arctic and Madeira. For the Azores deep-sea samples, however, up to five actinobacterial genera were detected in at least half of the samples analyzed, alongside *Illumatobacter* and *Sva0996 marine group*. The actinobacterial *Sva0996 marine group* has been detected in various marine sediments, including shelf sediments, tidal sediments (Miksch et al., 2021) and deep-sea sediments (Raggi et al., 2020). Similarly, the genus *Ilumatobacter* was described to be predominant in coastal surface sediments of the Mediterranean Sea (Duran et al., 2015) and of the Pacific Ocean (Choi et al., 2016), and it was also reported as one of the main taxa in deep-sea sediments of the Indic Ocean (Chen et al., 2016). On the other hand, many of the actinobacterial genera exclusively detected in the Azores deep-sea sediments (e.g., *Cutibacterium* and *Garnerella*) have never been described so far as being associated to deep-sea sediments.

The culture-dependent methods employed in our study led to the isolation of 44 actinobacterial strains, which were retrieved from deep-sea samples that suffered different pre-treatment schemes. As the deep-sea samples collected in the different geographical regions were not processed in the same time period, the distinct pre-treatments applied were a reflection of the experience acquired during their analysis, having in mind the limitation of non-actinobacterial growth and the maximization of growth of actinobacterial strains.

The retrieved actinobacterial isolates were affiliated with nine genera. Among the most common genera identified in our work, *Brachybacterium*, *Microbacterium*, and *Brevibacterium* were also previously isolated from deep-sea sediments (Zeng et al., 2010; Zhang et al., 2014; Prieto-Davó et al., 2016). The genus *Micrococcus*, isolated only from the Arctic samples, was also retrieved by Zhang et al. (2014) from marine sediments from the same region. Other studies report the isolation of members of this genus from Mediterranean deep-sea sediments (Jroundi et al., 2020) and from the Mariana Trench in the Pacific (Pathom-Aree et al., 2006). Regarding the genera *Leucobacter*, *Dietzia*, and *Rhodococcus*, detected in less abundance in our samples, these have not been reported so far in deep-sea sediments, although they have been detected in shallow marine sedimentary deposits (Saimmai et al., 2012; Hackbusch et al., 2020).

In our study, nine actinobacterial genera were identified using culture-dependent methods, while metabarcoding analysis, as a culture-independent method, led to the identification of 20 genera. Overall, only five genera were retrieved by both methods (*Brevibacterium*, *Dietzia*, *Microbacterium*, *Micrococcus*, and *Streptomyces*). Curiously, according to our metabarcoding data, the genera *Streptomyces*, *Dietzia*, and *Brevibacterium* were only detected in the Azores sediments, while these genera were isolated from the Arctic and Madeira sediments (A81, A82, and MA3). One possible explanation for this could lie in the fact that these genera were present in low abundance in the libraries constructed for metabarcoding analysis, but the culture conditions used in our study were suitable for their isolation. In addition, the DNA extraction methodology and biased primer amplification can have also caused differences in abundances, resulting in the underrepresentation of some genotypes in the environmental sample (Zhang et al., 2014). The fact that our rarefaction curve reached saturation suggests that there was a high recovery of bacterial diversity through the culture-independent approach. This diversity was not observed through culture-dependent method, which is in agreement with the well-known difficulty in isolating some genera under laboratory conditions. The combined use of culture-dependent and -independent approaches was essential to provide a better characterization of the actinobacterial community of our deep-sea sediment samples (Prieto-Davó et al., 2016; Anguita-Maeso et al., 2020).

Antibiotic resistance is a growing concern worldwide and, in this respect, screening microorganisms from unexplored regions, like the deep-sea, is highly important to boost the discovery of new antimicrobial compounds (Fiedler et al., 2005; Bredholt et al., 2008). In our study, the antimicrobial screening conducted with the extracts obtained from the isolated actinobacterial strains revealed two *Streptomyces*-derived extracts active against *C. albicans*. Some compounds exhibiting antifungal activity against *C. albicans* were already isolated from deep-sea sediments-associated *Streptomyces*, like Streptolactams A and C, polyene macrolactams with MIC values of 10.4 and 16.1 μM, respectively (Wang et al., 2020), and Tunicamycin E, an antibiotic with moderate antifungal activity, with MIC values between 2 and 32 μg/ml (Zhang et al., 2020). Comparing the MIC values of our extracts (7.81 and 15.62 μg/ml, respectively) with those of the previously mentioned compounds, suggests that our extracts exhibit potent antifungal activity.

Microorganisms have been the source of many chemotherapeutics used in cancer treatment, and amongst them, actinobacteria are one of the most promising sources (Manivasagan et al., 2014). Our results showed eight actinobacterial extracts (derived from *Rhodococcus*, *Streptomyces*, *Brevibacterium*, *Microbacterium*, and *Brachybacterium* strains, isolated from both the Arctic and Azores) with anticancer activity against at least of the tested cancer cell lines. Some of these extracts have the particularity of inhibiting only the cancer cell lines tested and not the non-cancer cell line, suggesting a selective anticancer activity. The anticancer potential of deep-sea *Streptomyces* strains has been demonstrated in previous studies carried out in sediments from the Arctic and the Madeira Archipelago, in which strains of this genus exhibited activity against the human lung cancer cell line and the human colon carcinoma cell line (Prieto-Davó et al., 2016; Dhaneesha et al., 2017). A vast number of natural products with anticancer activity has been reported for *Streptomyces* strains isolated from deep-sea sediments, such as Grincamycins B–F, C-glycoside angucyclines with anticancer activity against a wide range of cancer cell lines (Huang et al., 2012), Ammosamides A-B, anticancer agents with capacity to decrease the viability of the colon carcinoma cancer cell line HCT-116 (Hughes et al., 2009), and Spiroindimicins A–D, bisindole alkaloids with moderate cytotoxicity against various cancer cell lines. In terms of anticancer activity exhibited by deep-sea associated actinobacteria of the genus *Microbacterium*, only two compounds with cytotoxic activity have been described, the Microbacterins A and B that were isolated from the deep sea *Microbacterium sediminis* YLB-01 (Liu et al., 2015). As far as we know, no compounds with anticancer activity have been reported for deep-sea actinobacterial strains of the genera *Brevibacterium*, *Brachybacterium*, and *Rhodococcus*.

Very few studies have targeted the potential of deep-sea actinobacteria to produce anti-inflammatory compounds (Manivasagan et al., 2014), in spite of actinobacteria isolated from marine intertidal sediments (Park et al., 2006), mangroves (Gomathi and Gothandam, 2019) and macroalgae (Braña et al., 2015) having demonstrated anti-inflammatory potential. In our study, 15 actinobacterial extracts exhibited promising anti-inflammatory activity, with some of these extracts being derived from strains affiliated with genera that previously demonstrated this activity, like *Brevibacterium* (Srilekha et al., 2017) and *Brachybacterium* (Al-Halbosiy et al., 2018). On the other hand, we could not find studies reporting anti-inflammatory activity in actinobacteria associated to the genera *Microbacterium* and *Rhodococus* for any kind of environment, so, to the best of our knowledge, our study is the first one linking these genera to anti-inflammatory activity. Taking into account the scarcity of studies on the anti-inflammatory potential of actinobacteria and associated secondary metabolites, the study of the metabolic profile of each bioactive strain will be highly relevant for the discovery of new molecules with anti-inflammatory action. The fact that our extracts with anti-inflammatory activity are derived from actinobacteria isolated from extreme and underexplored environments further potentiates the chance of discovering new metabolites.

Metabolomic profiling offers a unique approach to find new natural products with biotechnological interest (Abdelrahman et al., 2022). In this study, we investigated the metabolic profile of all bioactive extracts obtained from the deep-sea actinobacterial strains. To obtain an overall knowledge of the metabolic profile, three dereplicator tools were employed. In a first analysis, using DEREPLICATOR, the compounds Surugamides A, B and D were found to be associated with only two extracts from *Streptomyces* isolated from the Madeira and Arctic regions. Surugamides are cyclic octapeptides, isolated from a marine-derived *Streptomyces* retrieved from deep-sea sediments collected at Kinko Bay, in Japan. These compounds were shown to have cytotoxic activity by inhibiting cathepsin B (Takada et al., 2013), however, this activity was not observed in our study, since the actinobacterial extracts did not show cytotoxic activity against the tested cancer cell lines. In addition, cyclic octapeptides are considered uncommon among bacterial secondary metabolism (Takada et al., 2013), nonetheless it has been described that the biosynthetic gene cluster responsible for their production are widely distributed in marine *Streptomyces* (Takada et al., 2013; Almeida et al., 2019).

According to the second analysis, that employed DEREPLICATOR VarQuest, it was possible to match our MS/MS date with analogues of known peptidic natural products using GNPS. One of the matches obtained were Phepropeptins A and D, which were found in the crude extract of *Microbacterium* sp. DS1_10.4, from Azores. These compounds, isolated from *Streptomyces* sp. MK600-cF7, are cyclic hexapeptides exhibiting anticancer activity (Sekizawa et al., 2001) and may explain this same activity observed in our extract.

In the other bioactive actinobacterial crude extracts, analogues of known lipopeptide antibiotics, such as cyclic octapeptide (Champacyclin) and cyclic depsipeptides (Amphomycin, Antibiotic A54145, Antibiotic A0341A, Daptomycin, Enduracidin C, Glycinocin B, Skyllamycin A-B and Telomycin) were detected. All these lipopeptide antibiotics were isolated from several *Streptomyces* strains and are known to exhibit activity against Gram-positive bacteria (Whaley et al., 1966; Boeck et al., 1990; Robbel and Marahiel, 2010; Yin et al., 2010; Pesic et al., 2013; Yang et al., 2014; Fu et al., 2015; Corcilius et al., 2017; Bracegirdle et al., 2021). However, the previously referred extracts did not exhibit antimicrobial activity that could be explained by the presence of these lipopeptide antibiotics.

The last analysis was conducted with DEREPLICATOR+, which generated the highest number of hits as this analysis, besides improving the annotation of peptide natural products, also allows the annotation of polyketides and terpenes (Mohimani et al., 2018). One of the hits of this analysis was the compound Mycolactone F, which was only found in the crude extract of *Streptomyces* sp. MA3_2.13 and may account for the observed anticancer activity. Mycolactone F is a polyketide-derived macrolide produced by the pathogen *Mycobacterium marinum* exhibiting cytotoxic, anti-inflammatory and immunosuppressive activities (Ranger et al., 2006). The other matches obtained with DEREPLICATOR+, included antifungal compounds (Dermostatin A-B, Monensin M1-M2, Flavofungin II, Vacidin A, Langkolide and Deferoxamine), antibiotics (Erythromycin, Landomycin A, Piericidins, A54556, H668, YL02107Q-A, and Concanamycin B) and anticancer agents (Antimycin A, Norcadamine; Supplementary Table S2), which may explain the activities observed in our bioactive actinobacterial crude extracts.

The overall metabolomic profile of our marine actinobacterial extracts revealed a diversity of natural products similar to other metabolomic studies in deep-sea sediments (Tangerina et al., 2020; Abdelrahman et al., 2022). However, dereplication analysis showed matches to several natural products for which bioactivities were not observed in our extracts. This can be due to the relative amount of secondary metabolites present in the actinobacterial extracts. As mass spectrometry is a very accurate technique, even compounds with low abundances can be detected and, consequently, noted as hits in dereplication (Tangerina et al., 2020). Thus, the lack of activity observed may be due to the low concentration of these compounds in the crude extracts. On the other hand, dereplication of the bioactive actinobacterial extracts showed that some of the observed activities may result from new compounds, since hits to natural products accounting for these bioactivities were not obtained.

As the GNPS platform provides annotation comparing fragmentation patterns, it is possible to detect not only compounds present in a sample and but also their analogues that have similar fragments in the mass spectrum (Tangerina et al., 2020). Therefore, the construction of a molecular network allows grouping together sets of spectra of related molecules to form clusters, even when the spectra do not correspond to any known compound (Wang et al., 2016). For the crude extract derived from *Streptomyces* sp. MA3_2.13, three different clusters with major compounds m/z 297.17, m/z 387.180 and m/z 543.369 were found, which were associated with methyl 2-(3,5-dimethyl-2-oxocyclohexyl)tetrahydro-6-oxo-2H-pyran-4-acetate, furaquinocin H and Milbemycin β2, respectively. Cycloheximide derivative, methyl 2-(3,5-dimethyl-2-oxocyclohexyl)tetrahydro-6-oxo-2H-pyran-4-acetate, was isolated from the marine-derived *Streptomyces* sp. h-119 and does not show antifungal activity like Cycloheximide (Dou et al., 2015). The antibiotic Furaquinocin H was isolated from *Streptomyces* sp. KO-3988 and exhibits cytocidal activity against human cervical adenocarcinoma (HeLa S3) and murine melanoma (B16; Ishibashi et al., 1991). Milbemycin β2 shows antibiotic and anthelmintic properties and is produced by *Streptomyces* sp. B-41-146 (Mishima et al., 1975).

The additional cluster found in the extract of *Microbacterium* sp. DS3_6.1 (major compound m/z 694.878), did not result in any matches with natural products present in the databases, so it probably corresponds to a new natural product. Furthermore, as only single-strain clusters were analyzed in detail, there might be additional chemical novelty when analyzing the set of crude extracts. These results show the importance of combining different techniques of metabolomic studies with bioactive assays for strain prioritization.

## 5. Conclusion

This study assessed the actinobacterial diversity of deep-sea sediments from the Arctic (Arctic-Mid-Ocean Ridge) and Atlantic (Azores and Madeira) oceans, by simultaneously employing culture-dependent and -independent methods, and evaluated the bioactive potential of the actinobacterial strains isolated from those samples. Our work constitutes one of the first integrated assessments of the diversity of actinobacteria and their biosynthetic potential in these underexplored regions. Culture-independent studies revealed that the Actinobacteria phylum was more abundant in deep-sea sediments from the Arctic when compared to samples from the Atlantic Macaronesia region. This abundance was due to just a few taxa, namely belonging to the order *Actinomarinales* and to the families *Ilumatobactereaceae* and *Microtrichaceae*. On the other hand, deep-sea sediments from Madeira and Azores revealed a superior diversity of actinobacterial families, despite the lower overall incidence of the Actinobacteria phylum in these samples. Culture-dependent methodologies led to the recovery of a total of 44 actinobacterial isolates from the studied deep-sea sediment samples, which were distributed by nine genera. In terms of the bioactive potential of the isolated strains, our findings indicate that actinobacteria derived from deep-sea sediments are an important source of bioactive compounds. One of the highlights of our results was the significant anti-inflammatory activity of the tested actinobacterial extracts, which is particularly noteworthy since this bioactivity remains almost totally unreported for this phylum. The overall metabolomic profile of our deep-sea actinobacterial extracts revealed the presence of some metabolites associated to known natural products, but also possibly a new one since hits to natural products accounting for the observed bioactivity were not obtained. Our results thus demonstrate that bioprospecting actinobacteria in unexplored or underexplored environments, like the deep-sea, is a promising strategy for potentiating the discovery of new molecules with pharmaceutical applications. Further, characterizing the diversity of these poorly known sites is essential if robust resource management measures are to be developed and implemented.

## Data availability statement

The data presented in the study are deposited in the https://www.ebi.ac.uk/ena, accession number PRJEB59507; and in the https://www.ncbi.nlm.niah.gov/genbank/, accession number OQ363614-OQ363657.

## Author contributions

IR and EA carried out the isolation of actinobacteria and taxonomic identification. JA performed anti-inflammatory assays. IR carried out the antimicrobial and anticancer assays, LC–MS, and molecular networking/dereplication. PL supervised all chemical analyses. DA and MT performed bioinformatics analysis of metabarcoding data and AM curated this analysis. AH supplied the Arctic sediments samples. RU supervised the anticancer and anti-inflammatory assays. MC and AM designed and supervised the whole study. IR and JA led the writing of the manuscript with contributions from all the co-authors. All authors contributed to the article and approved the submitted version.

## Funding

This work was funded by the project ATLANTIDA—Platform for the monitoring of the North Atlantic Ocean and tools for the sustainable exploitation of the marine resources (reference NORTE-01-0145-FEDER-000040), supported by the North Portugal Regional Operational Programme (NORTE2020), and through the European Regional Development Fund (ERDF). It was also partially funded by the strategic Funding UIDB/04423/2020 and UIDP/04423/2020 through national funds provided by FCT and FEDER. IR acknowledges FCT for the PhD grant SFRH/BD/136357/2018. MC wishes to acknowledge the CEEC program supported by FCT (CEECIND/02968/2017), Fundo Social Europeu, and Programa Operacional Potencial Humano. AH was supported by funds from FCT/MCTES granted to CESAM (UIDP/50017/2020 + UIDB/50017/2020 + LA/P/0094/2020). The samples from the Arctic Mid-Ocean Ridge were collected in the framework of the project MarMine, funded by the Research Council of Norway (Project No. 247626/O30). The samples from the Azores were collected during the oceanographic campaign EMPC/PEPC/LUSO2016 organized by the Portuguese Task Group for the Extension of the Continental Shelf. The samples from Madeira were collected during the oceanographic mission SEDMAR 1/2017, conducted by the Portuguese Hydrographic Institute.

## Conflict of interest

The authors declare that the research was conducted in the absence of any commercial or financial relationships that could be construed as a potential conflict of interest.

## Publisher’s note

All claims expressed in this article are solely those of the authors and do not necessarily represent those of their affiliated organizations, or those of the publisher, the editors and the reviewers. Any product that may be evaluated in this article, or claim that may be made by its manufacturer, is not guaranteed or endorsed by the publisher.

## Footnotes

^1^ https://blast.ncbi.nlm.nih.gov/Blast.cgi

^2^ https://www.ezbiocloud.net/
