# Corrigendum: Actinobacteria from Arctic and Atlantic deep-sea sediments—Biodiversity and bioactive potential

**DOI:** 10.3389/fmicb.2023.1252355

**Published:** 2023-07-18

**Authors:** Inês Ribeiro, Jorge T. Antunes, Diogo A. M. Alexandrino, Maria Paola Tomasino, Eduarda Almeida, Ana Hilário, Ralph Urbatzka, Pedro N. Leão, Ana P. Mucha, Maria F. Carvalho

**Affiliations:** ^1^CIIMAR - Interdisciplinary Centre of Marine and Environmental Research, University of Porto, Porto, Portugal; ^2^School of Medicine and Biomedical Sciences (ICBAS), University of Porto, Porto, Portugal; ^3^Department of Environmental Health, School of Health, Polytechnic of Porto, Porto, Portugal; ^4^Department of Biology, FCUP - Faculty of Sciences of the University of Porto, Porto, Portugal; ^5^Centre for Environmental and Marine Studies and Department of Biology, University of Aveiro, Aveiro, Portugal

**Keywords:** actinobacteria, antimicrobial, anti-cancer, anti-inflammatory, deep-sea sediments, metabarcoding

In the published article, there was an error in the **Funding** statement. The statement “This work was funded by the project ACTINODEEPSEA POCI-01-0145-FEDER-031045 (PTDC/BIA-MIC/31045/2017) financed by FEDER—Fundo Europeu de Desenvolvimento Regional funds through the COMPETE 2020—Operacional Programme for Competitiveness and Internationalization (POCI), Portugal 2020, and by Portuguese funds through FCT—Fundação para a Ciência e a Tecnologia/Ministério da Ciência, Tecnologia e Ensino Superior” was included, but is incorrect. The correct Funding statement appears below.

