# Supplementary material for: Actinobacteria from Arctic and Atlantic deep-sea sediments—Biodiversity and bioactive potential
Source: Front Microbiol. 2023 Mar 30;14:1158441. doi: 10.3389/fmicb.2023.1158441 (PMC10100589; doi:10.3389/fmicb.2023.1158441)
Supplement: Supplementary file 1 [file Table_1.pdf]

Table S1 - Taxonomic identification of the actinobacterial isolates retrieved from analysed the deep-sea sediments and corresponding GenBank accession number.

Campaign	Geographical locations	Isolate	Closest identification*	Similarity (%)†	Sequence length (bp)	GenBank accession number
**MarMine Arctic Mid-Ocean Ridge (AMOR) 2016**	Artic Ocean	78.9	*Micrococcus yunnanensis*	99.64	1375	OQ363622
		78.10	*Micrococcus yunnanensis*	99.78	1378	OQ363620
		78.3	*Brachybacterium paraconglomeratum*	99.76	1269	OQ363621
		79_1.6	*Brachybacterium paraconglomeratum*	99.35	1378	OQ363628
		79_1.24	*Brevibacterium antiquum*	**98.61**	1323	OQ363625
		79_1.25	*Brevibacterium antiquum*	**98.42**	1391	OQ363626
		79_1.12	*Brevibacterium antiquum*	**98.61**	1373	OQ363623
		79_1.12 (A)	*Brachybacterium paraconglomeratum*	99.77	1327	OQ363624
		79_1.4	*Brachybacterium paraconglomeratum*	99.62	1301	OQ363627
		80_1.6	*Brevibacterium antiquum*	**98.49**	1392	OQ363630
		80_1.4	*Brachybacterium paraconglomeratum*	99.73	1273	OQ363629
		80_1.7	*Brachybacterium paraconglomeratum*	99.70	1326	OQ363631
		81_2.5	*Brevibacterium antiquum*	**98.22**	1400	OQ363633
		81_1.2	*Streptomyces* sp.	99.69	1290	OQ363632
		82_2.6	*Brevibacterium antiquum*	**98.35**	1392	OQ363637
		82_2.13	*Streptomyces* sp.	99.71	1379	OQ363636
		82_1.3	*Brachybacterium paraconglomeratum*	99.83	1310	OQ363634
		82_2.10	*Brachybacterium paraconglomeratum*	99.79	1288	OQ363635
		82_2.8	*Brachybacterium paraconglomeratum*	99.79	1331	OQ363638
		136.12	*Actinotalea* sp.	**97.85**	1395	OQ363614
		136.30	*Brevibacterium antiquum*	**98.54**	1367	OQ363619
		136.19	*Actinotalea ferrariae*	**98.40**	1370	OQ363617
		136.2	*Dietzia psychralcaliphila*	100	1362	OQ363618
		136.13	*Actinotalea subterranea*	**98.32**	1371	OQ363615
		136.15	*Brevibacterium antiquum*	**98.21**	1389	OQ363616

**IH mission SEDMAR 1/2017**	Madeira region	E147_1.1	*Brevibacterium salitolerans*	98.99	1394	OQ363651
		E147_1.8	*Brachybacterium paraconglomeratum*	99.69	1303	OQ363652
		MA3_2.11	*Brevibacterium sediminis*	99.93	1376	OQ36365
		MA3_2.14	*Streptomyces resistomycificus*	100	1391	OQ363656
		MA3 2.13	*Streptomyces* sp.	**97.37**	1405	OQ363655
		MA3_1.18	*Brachybacterium paraconglomeratum*	99.79	1300	OQ363653
		MA3_2.6	*Leucobacter komagatae*	100	1272	OQ363657
**EMEPC/PEPC/LUSO/2016**	Azores region	DS1_6	*Microbacterium testaceum*	99.33	1352	OQ363641
		DS1_10.2	*Microbacterium testaceum*	99.33	1352	OQ363639
		DS1_10.4	*Microbacterium testaceum*	99.34	1365	OQ363640
		DS3_6.1	*Microbacterium testaceum*	99.34	1368	OQ363645
		DS3_17.1	*Microbacterium testaceum*	99.25	1206	OQ363642
		DS3_17.2	*Microbacterium testaceum*	99.30	1285	OQ363643
		DS3_19.2	*Microbacterium testaceum*	99.34	1369	OQ363644
		DS4_2.1	*Microbacterium testaceum*	99.34	1365	OQ363647
		DS4_2.3	*Microbacterium testaceum*	99.35	1375	OQ363648
		DS4_3	*Rhodococcus* sp.	100	1167	OQ363650
		DS4_18	*Rhodococcus* sp.	100	1190	OQ363646
		DS4_20	*Rhodococcus yunnanensis*	99.83	1171	OQ363649

*According to 16S ribosomal RNA (Bacteria and Archaea type strains) database from NCBI BLAST.
†Values in bold indicate potential new species.
