# Supplementary material for: Actinobacteria from Arctic and Atlantic deep-sea sediments—Biodiversity and bioactive potential
Source: Front Microbiol. 2023 Mar 30;14:1158441. doi: 10.3389/fmicb.2023.1158441 (PMC10100589; doi:10.3389/fmicb.2023.1158441)
Supplement: Supplementary file 2 [file Table_2.pdf]

Table S2 - Unique Metabolite List for all bioactive actinobacterial extracts that correspond to annotated molecules produced by actinobacteria strains. This list features DEREPLICATOR, DEREPLICATOR VarQuest, and DEREPLICATOR+ annotations that are sorted by score, mass, compound name, adduct, and FDR %. For analysis of the results, a threshold p-value ≤ 10^{-10} for DEREPLICATOR and DEREPLICATOR VarQuest, and a very strict score value of 15 on DEREPLICATOR+ were selected to reduce false matches.

DEREPLICATOR

Strain ID	Compound Name	Score	p-Value	Peptide Mass	Spectrum Mass	Adduct	Peptide FDR %
82_2.13	Surugamide B	16	4.80E-48	897.61	898.61	M+H	0
MA3_2.14	Surugamide A	16	7.30E-48	911.62	912.63	M+H	0
MA3_2.14	Surugamide D	12	1.50E-35	897.61	898.61	M+H	0

DEREPLICATOR VARQUEST

Strain ID	Compound Name	Score	p-Value	Peptide Mass	Spectrum Mass	Adduct	Peptide FDR %
DS3_6.1	Amphomycin	10	3.30E-16	1289.65	703.395	M+2H	0
136_2	Antibiotic A0341A_3-Hydroxy	6	7.90E-14	1140.52	592.785	M+2H	0
136_2	Antibiotic A54145_3-Demethoxy	7	3.20E-15	1627.77	781.896	M+2H	0
79_1.6	Antibiotic A54145_3-Demethoxy	12	8.70E-19	1627.77	872.457	M+2H	0
79.4	Antibiotic A54145_3-Demethoxy,_3'''-deoxy	8	1.80E-18	1611.78	761.383	M+2H	0
79_1.6	Antibiotic A54145_3-Demethoxy,_3'''-deoxy	13	5.90E-21	1625.79	873.431	M+2H	0
79_1.6	Antibiotic A54145_3'''-Deoxy,_3-O-de-Me	12	6.50E-19	1627.77	845.42	M+2H	0
80_1.6	Antibiotic A54145A	14	1.20E-22	1643.77	844.421	M+2H	20
80_1.6	Antibiotic A54145D	13	5.50E-21	1657.78	828.925	M+2H	0
136_2	Antibiotic A54145F	6	5.10E-12	1629.75	801.911	M+2H	0
80_1.6	Antibiotic A54145F	11	1.50E-15	1629.75	872.457	M+2H	0
MA3_2.14	Champacyclin	11	8.00E-33	897.605	884.596	M+H	0
79_1.6	Daptomycin	10	2.70E-15	1619.71	847.912	M+2H	0
80_1.6	Enduracidin-C	10	2.60E-10	2380.97	797.084	M+3H	0
79.4	Glycinocin B	6	5.90E-14	1260.66	639.329	M+2H	0
DS1_10.4	Phepropeptin A	10	1.30E-26	682.442	764.469	M+H	0
DS1_10.4	Phepropeptin D	10	9.00E-27	730.442	778.485	M+H	0
80_1.6	Skyllamycin A	12	1.60E-18	1480.69	692.836	M+2H	0

80_1.6	Skyllamycin B	12	1.90E-19	1468.66	751.867	M+2H	0
82_2.13	Surugamide A	21	9.60E-58	911.621	982.634	M+H	0
MA3_2.14	Surugamide C	15	6.60E-44	897.605	878.643	M+H	0
MA3_2.14	Surugamide D	10	1.40E-26	897.605	458.332	M+2H	0
79_1.6	Telomycin	10	4.00E-17	1271.55	600.782	M+2H	0
136_2	Zelkovamycin	7	1.40E-16	779.306	426.719	M+2H	0

DEREPLICATOR+

Strain ID	Metabolite	Score	p-Value	Metabolite Mass	Spectrum MZ	Adduct	Metabolite FDR %
MA3 2.13	Antibiotic A54556D	16	NA	720.385	721.388	M+H	0
DS3_6.1	Antibiotic H668	18	NA	688.44	689.45	M+H	0
MA3 2.13	Antibiotic YL 02107Q-A	17	NA	850.493	851.505	M+H	0
82_2.13	Antimycin A	15	NA	478.195	479.203	M+H	0
MA3 2.13	Concanamycin B	17	NA	808.497	809.506	M+H	0
82_2.13	Deferoxamine	15	NA	658.463	659.47	M+H	0
MA3_2.14	Deferoxamine	16	NA	636.385	637.391	M+H	0
136.30	Dermostatin A	27	NA	720.445	721.456	M+H	0
136.30	Dermostatin B	33	NA	734.461	735.473	M+H	0
MA3 2.13	Desferrioxamine G	16	NA	618.359	619.367	M+H	0
DS1_10.4	Erythromycin	20	NA	717.466	718.47	M+H	0
DS1_10.4	Flavofungin II	15	NA	664.419	665.424	M+H	0
DS3_6.1	Flavofungin II	18	NA	664.419	665.425	M+H	0
DS4.3	Landomycin A	15	NA	1068.46	535.236	M+2H	0
136.30	Langkolide	16	NA	1446.75	724.381	M+2H	0
79_1.6	Malacidin A	19	NA	1248.62	625.315	M+2H	0
79.4	Monensin M1	15	NA	672.445	673.45	M+H	0
79_1.12(A)	Monensin M1	18	NA	672.445	673.45	M+H	0
78.3	Monensin M2	17	NA	688.44	689.449	M+H	0
79_1.12	Monensin M2	18	NA	688.44	689.451	M+H	0

MA3 2.13	Mycolactone F	16	NA	714.507	715.514	M+H	0
79_1.6	Nocardamine	16	NA	584.353	585.361	M+H	0
82_2.13	Nocardamine	21	NA	584.353	585.361	M+H	0
DS4.20	Piericidin	15	NA	385.262	386.269	M+H	0
DS4.20	Piericidin a1	15	NA	415.272	416.279	M+H	0
82_2.13	Proferrioxamine A1	17	NA	546.338	547.346	M+H	0
MA3 2.13	VacidinA	15	NA	1112.57	557.294	M+2H	0
